# Comparative Analysis of the Microbial Community Profiles of Sichuan and Guizhou Smoke-Cured Sausages Using a High-Throughput Sequencing Approach

**DOI:** 10.3390/microorganisms13051096

**Published:** 2025-05-08

**Authors:** Xiangyong Zeng, Chaoyang Wei, Dounan Li, Wentao Cao, Qiang Lin

**Affiliations:** 1 School of Liquor and Food Engineering, Guizhou University, Guiyang 550025, China; xyzeng1@gzu.edu.cn (X.Z.); cywei@gzu.edu.cn (C.W.); dnli@gzu.edu.cn (D.L.); 2 Guizhou Provincial Key Laboratory of Fermentation and Biopharmacy, Guizhou University, Guiyang 550025, China; 3 Chengdu Institute of Biology, Chinese Academy of Sciences, Chengdu 610041, China

**Keywords:** smoke-cured sausage, spontaneous fermentation, microbial community, high-throughput sequencing, *Cobetia*

## Abstract

Autochthonous microorganisms play critical roles in shaping the quality of Chinese sausages and may be influenced by local climate and/or processing conditions. The present study aimed to reveal the interprovincial differences in microbial community between Sichuan and Guizhou sausages, as well as driving factors based on high-throughput sequencing and bioinformatic analysis. The results indicated that *Cobetia*, *Debaryomycetaceae*, *Kurtzmaniella*, and *Candida zeylanoides* served as biomarkers for Sichuan sausages. In contrast, *Enterococcus*, unclassified Cyanobacteriales, *Lactobacillales*, *Aspergillus vitricola*, *Mortierella*, *Fusarium,* and *Penicillium* were identified as biomarkers for Guizhou sausages. Furthermore, salt content and moisture level showed positive correlations with *Cobetia, Staphylococcus*, *Debaryomyces*, and *Kurtzmaniella,* mainly found in Sichuan sausages. Conversely, pH and water activity (Aw) were positively associated with potential pathogenic bacteria (e.g., *Vibrio*, *Cyanobacteria*, *Enterococcus*, and *Aeromonas*) and fungi (e.g., *Aspergillus*, *Fusarium*, and *Penicillium*), which were mainly distributed in Guizhou sausages. Notably, microbial composition discrepancies between Sichuan and Guizhou sausages were primarily driven by processing conditions rather than regional climate factors. Collectively, these findings provide valuable insight for developing novel specific starters.

## 1. Introduction

Sausages are traditional fermented meat products widely favored by consumers globally. In European countries, sausages such as salami are typically produced using starters composed of functional microbes and are widely distributed in Italy, Spain, and Germany [1]. In contrast, Chinese sausages are traditionally prepared by mixing pork lean/fat meat with ingredients such as salt, sugar, pepper powder, chili powder, and baijiu. The mixture is then stuffed into a natural casing (e.g., small intestine) and subjected to smoke curing and spontaneous fermentation [2]. Accordingly, autochthonous microorganisms—primarily derived from raw materials, processing tools, human skin, and the environment—colonize the meat matrix due to its nutrient-rich composition. These microorganisms excrete hydrolases to degrade carbohydrates, proteins, and lipids, generating flavor precursors and aromatic compounds. Meanwhile, they effectively inhibit undesired microbiota and mitigate the accumulation of harmful metabolites. Collectively, microbial communities play a critical role in determining the flavor, quality, and safety of sausages during spontaneous fermentation [3]. Thus, unveiling the microbial composition of smoke-cured sausages is essential for improving their quality and safety.

Both culture-dependent and culture-independent approaches have been extensively used to characterize microbial diversity in fermented sausages. Initially, functional microorganisms in sausages were isolated and identified using traditional cultivation methods. *Staphylococcus* species such as *S. succinus* and *S. xylosus* were largely isolated during the maturation stage of Italian sausages, as well as lactic acid bacteria (LAB) [4]. Although culture-dependent methods have identified numerous functional microbes, these methods are limited by low reliability, accuracy, and efficiency. Moreover, rare species and unculturable microorganisms often evade isolation, hindering comprehensive profiling of authentic microbial communities [5,6]. Subsequently, polymerase chain reaction–denaturing gradient gel electrophoresis (PCR-DGGE) was employed to analyze microbial profiles in fermented sausages from Italy [7,8], Portugal [9], Argentina [10], and China [11,12]. High-throughput sequencing (HTS) technology has since emerged as a superior tool, offering enhanced accuracy, throughput, robustness, and speed for elucidating microbial community structure and succession in complex ecosystems. HTS-based studies have provided in-depth insights into microbial communities of fermented meat products, including air-dried beef, sausage, and yak jerky, etc. [13,14,15].

Sichuan and Guizhou provinces, located in southwestern China, have traditional practices of homemade smoke-cured sausage production during winter. Sichuan sausages are typically prepared with a lean-to-fat pork ratio of 1.5:1, supplemented with 50–60 g/kg NaCl, 2 g/kg sugar, 5 g/kg chili powder, 50 mg/kg pepper powder, and baijiu (Chinese liquor). In contrast, Guizhou sausages use a lean-to-fat ratio of 1:1, 20–30 g/kg NaCl, 2 g/kg sugar, 50 mg/kg pepper powder, and baijiu. Both sausages undergo smoke curing and air-drying, differing primarily in processing duration. Sichuan sausages are initially smoke-cured for one day using cypress leaf, orange peel, and peanut shell, followed by air-drying for over 20 days. Guizhou sausages, however, are suspended in kitchens and exposed to wood smoke during daily cooking for approximately one month. Sensorily, Sichuan sausages are characterized by spiciness and saltiness, whereas Guizhou sausages exhibit a mild sourness. These taste discrepancies are greatly influenced by microbial composition and processing conditions. Although prior studies have reported microbial communities in Sichuan sausages using HTS [12,14,16], comparative analyses of interprovincial microbial profiles (Sichuan vs. Guizhou) remain scarce [17]. Additionally, the factors driving microbial compositional differences between these sausages are poorly understood.

Therefore, this study aimed to (1) compare microbial compositions of smoke-cured sausages at the interprovincial level and (2) identify key factors driving microbial community divergence. The findings provide critical insights for developing novel specific starters to optimize sausage quality and safety.

## 2. Materials and Methods

### 2.1. Sausages Collection

**Figure 1:**
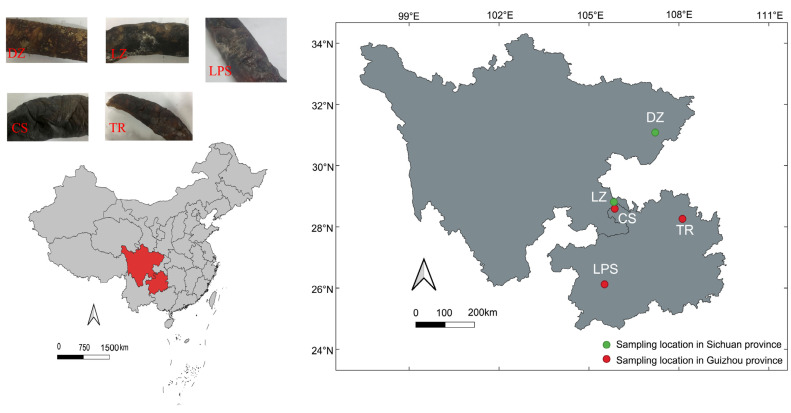
The appearances and sampling locations of five smoke-cured sausage samples that were collected from Sichuan and Guizhou provinces in southwest China. DZ: Dazhou; LZ: Luzhou; CS: Chishui; LPS: Liupanshui; TR: Tongren. Green and read circles denoted Sichuan and Guizhou sausage samples, respectively.

A total of five smoke-cured sausage samples were collected in March 2022. Two samples (labeled DZ and LZ) originated from Dazhou (107.2° E, 31.1° N) and Luzhou (105.8° E, 28.8° N) in Sichuan Province. The three remaining samples—CS, LPS, and TR—were collected from Chishui (105.9° E, 28.6° N), Liupanshui (105.5° E, 26.1° N), and Tongren (108.1° E, 28.3° N) in Guizhou Province, respectively. The geographical locations of the sampling sites are illustrated in Figure 1. All samples were stored in an ice box and transported to the laboratory within 24 h. Subsequently, the sausages were frozen at −20 °C for physicochemical parameter detection and microbial community profiling.

### 2.2. Detection of Physicochemical Parameters

The moisture content of sausages was detected by a portable moisture meter (SFY-30, Guanya, Shenzhen, China) according to the manufacturer’s instructions. Water activity (Aw) was determined with a water activity instrument (HD-4B, Huake Apparatus Co., Shenzhen, China) at 25 °C. Briefly, 3 g of a homogenized sausage sample was evenly distributed in the bottom of the water activity apparatus. The pH values of sausages were recorded using a portable pH meter (Testo 205, Lenzkirch, Germany), by directly inserting the probe into the samples. For salt content, 1 g of sausage was homogenized with 9 mL of distilled water, and salinity was quantified using a salt meter (Pal-saltprobe, ATAGO Co., Fukuoka City, Japan). These four physicochemical parameters were measured in triplicate.

### 2.3. DNA Extraction, PCR Amplification, and High-Throughput Sequencing

About 1 g of sausage from each of the 15 duplicates was flash-frozen in liquid nitrogen and then ground into powder. Genomic DNA was extracted using the TGuide S96 Magnetic Beads Soil/Fecal DNA extraction kit (TIANGEN, DP812, Beijing, China) according to the manufacturer’s instructions. Bacterial 16S rRNA gene V3–V4 regions were amplified with primers 338F/806R, and fungal ITS1 regions were amplified with primers ITS1F/ITS2 via a two-step PCR approach. First PCR (10 μL) consisted of 5–50 ng template DNA, 0.3 μL each primer, 5 μL KOD FX Neo Buffer, 2 μL dNTP (2 mM), 0.2 μL KOD FX Neo, and nuclease-free water to 10 µL. The PCR conditions were as shown below: initial denaturation at 95 °C for 5 min, 25 cycles of 95 °C for 30 s, 50 °C for 30 s, and 72 °C for 40 s, final extension at 72 °C for 7 min. The second PCR reaction system (20 μL) was as follows: 5 μL initial targeting PCR products, 2.5 μL MPPI-a/MPPI-b (2 μM), 10 μL 2 × Q5 High-Fidelity Master Mix. The PCR reaction conditions were as follows: initial denaturation at 98 °C for 30 s, 10 cycles of 98 °C for 10 s, 65 °C for 30 s, and 72 °C for 30 s, final extension at 72 °C for 5 min. The agarose gel electrophoresis (1.8% w/v) was used to check the target PCR products. After checked and quantified, the targeted products were used to build a cloning library. The library quality was assessed on the Qubit@2.0 Fluorometer (Thermo Scientific, Waltham, MA, USA). Finally, paired-end sequencing was performed on the Illumina novaseq 6000 platform (Illumina, San Diego, CA, USA).

### 2.4. Bioinformatics Analysis

Raw sequences of bacterial 16S rRNA (V3-V4) and fungal ITS1 regions were firstly filtrated by Trimmomatic (version 0.33). Removal of primer sequences was performed by Cutadapt (version 1.9.1) to obtain clean reads. Then, the paired-end reads obtained were assembled by USEARCH (version 10), followed by Chimera removal using UCHIME (version 8.1). Sequences with ≥97% similarity were clustered into the same operational taxonomic units (OTUs). Taxonomic annotation was performed against the SILVA (v138) and UNITE (v8.3) databases for bacteria and fungi, respectively. Alpha diversity (Chao1, Shannon, Simpson, and PD whole tree, etc.) and beta diversity analyses were performed using QIIME software (Version 1.7.0). Abundance analysis, principal component analysis (PCA), linear discriminant analysis effect size (LEfSe), and Spearman’s correlation heatmaps were visualized using R package (v2.15.3). Raw bacterial and fungal DNA sequences were deposited in the NCBI Sequence Read Archive (SRA) under accession number: PRJNA870249.

## 3. Results

### 3.1. Physicochemical Parameters of Sausages from the Sichuan and Guizhou Region

The pH, moisture content, Aw, and salt content of the sausages were detected and are shown in Table 1. The pH value of the five fermented sausages ranged from 5.51 to 6.22. As expected, sample TR showed the lowest pH value (5.51). Although no linear relationship was observed between Aw and moisture content, a positive correlation was noted. Specifically, sample CS (14.15% moisture) displayed the lowest Aw (0.761), whereas samples with >20% moisture showed Aw > 0.8. Notably, the NaCl content in two Sichuan sausages (DZ and LZ) exceeded 5%, roughly twice that of Guizhou sausages.

### 3.2. α-Diversity Analysis

Representative OTUs obtained from the Illumina Novaseq platform were used to characterize the bacterial and fungal diversities of these 15 sausage duplicates. Abundance-based indices (ACE and Chao1), diversity indices (Shannon and Simpson), and phylogenetic diversity (PD whole tree) are summarized in Table 2. Usually, Shannon and Simpson indices reflect microbial community diversity and evenness, while Chao1 and ACE estimate species richness. For bacterial communities, sample DZ exhibited the lowest ACE, Chao1, Shannon, and Simpson values, indicating reduced diversity and high evenness. In contrast, the remaining four samples displayed bacterial Shannon indices >4.30, suggesting higher diversity. Notably, fungal diversity (Shannon index) surpassed bacterial diversity in sample TR. Furthermore, the fungal Shannon indices were highest in TR and CS, whereas DZ, LZ, and LPS showed minimal fungal diversity. The PD whole-tree values revealed distinct genetic relationships: the bacterial communities in DZ and LPS exhibited the simplest and most complex genetic profiles, respectively. Similarly, the fungal PD values indicated simplified genetic relationships in DZ and LZ, contrasting with the complex profiles observed in TR and CS.

### 3.3. Abundance Analyses of the Bacterial and Fungal Community Composition of Sausages at the Phylum and Genus Levels

**Figure 2:**
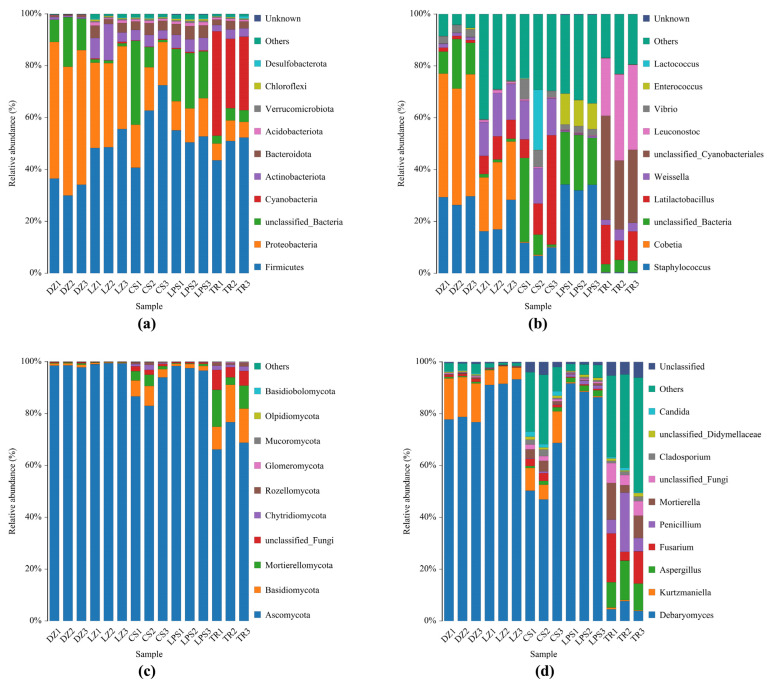
Abundance analysis of bacteria (**a**,**b**) and fungi (**c**,**d**) obtained from five sausage samples at the phylum level and genus level. DZ: Dazhou; LZ: Luzhou; CS: Chishui; LPS: Liupanshui; TR: Tongren. These five sausage samples were measured in triplicate.

Following taxonomic annotation, the relative abundances of the top 10 abundant microbial phyla and genera are illustrated in Figure 2. At the phylum level, *Firmicutes*, *Proteobacteria*, *Cyanobacteria*, *Actinobacteriota*, and *Bacteroidota* were the predominant phyla. *Firmicutes* and *Proteobacteria* collectively accounted for >60% of the total abundance (reaching up to 90% in most samples), except in CS1 and TR1. Notably, *Firmicutes* exhibited a lower relative abundance (~30%) in the Sichuan sample DZ, whereas *Proteobacteria* accounted for more than 50%. In contrast, the three Guizhou sausage samples displayed an inverse pattern, with *Firmicutes* significantly surpassing *Proteobacteria* in abundance. Intriguingly, the phylum *Cyanobacteria* were exclusively detected in sample TR with a relative abundance >30%, which exhibited the lowest pH and NaCl values (Figure 2a).

At the genus level, the predominant bacterial genera in both Sichuan and Guizhou sausages included *Staphylococcus*, *Cobetia*, unclassified Cyanobacteriales, *Vibrio*, and *Enterococcus*, and lactic acid bacteria (LAB; *Latilactobacillus*, *Leuconostoc*, *Weissella*, and *Lactococcus*) were found. The genus *Staphylococcus* was present in all samples except TR, accounting for approximately 15% in LZ and 40% in LPS. Meanwhile, unclassified Cyanobacteriales and LAB (especially *Leuconostoc*) predominated in TR, which exhibited the lowest pH and saline content. Intriguingly, halophilic *Cobetia* was exclusively identified in high-salt Sichuan sausages, constituting >45% of DZ and ~20% of LZ. Potential pathogens such as unclassified Cyanobacteriales, *Vibrio*, *Enterococcus* were mainly distributed in the Guizhou samples TR, CS, and LPS (Figure 2b).

As regards fungal diversity, *Ascomycota*, *Basidiomycota*, and *Mortierellomycota* were the dominant phyla in sausages. The phylum *Ascomycota* accounted for 96% of samples DZ, LZ, and LPS, even near 100% in sample LZ. Additionally, the total proportion of both phyla *Mortierellomycota* and *Basidiomycota* was 15–25% in sample TR, though *Ascomycota* was still the dominant fungal phylum (Figure 2c). At the genus level, *Debaryomyces*, *Kurtzmaniella*, *Aspergillus*, *Penicillium*, *Fusarium*, and *Mortierella* were the dominant genera in all sausage samples. As expected, the most abundant genera, *Debaryomyces* and *Kurtzmaniella*, accounted for over 90% of samples DZ and LZ with an elevated saline content. In contrast, the genus *Debaryomyces* in sample TR was the least abundant among all sausage samples, reflected by its <8% proportion. Accordingly, the abundances of the genera *Aspergillus*, *Penicillium*, *Fusarium*, and *Mortierella*, and some potential pathogenic fungi were high in sample TR, followed by sample CS (Figure 2d).

### 3.4. Principle Component Analysis (PCA) and Heatmap Analysis

**Figure 3:**
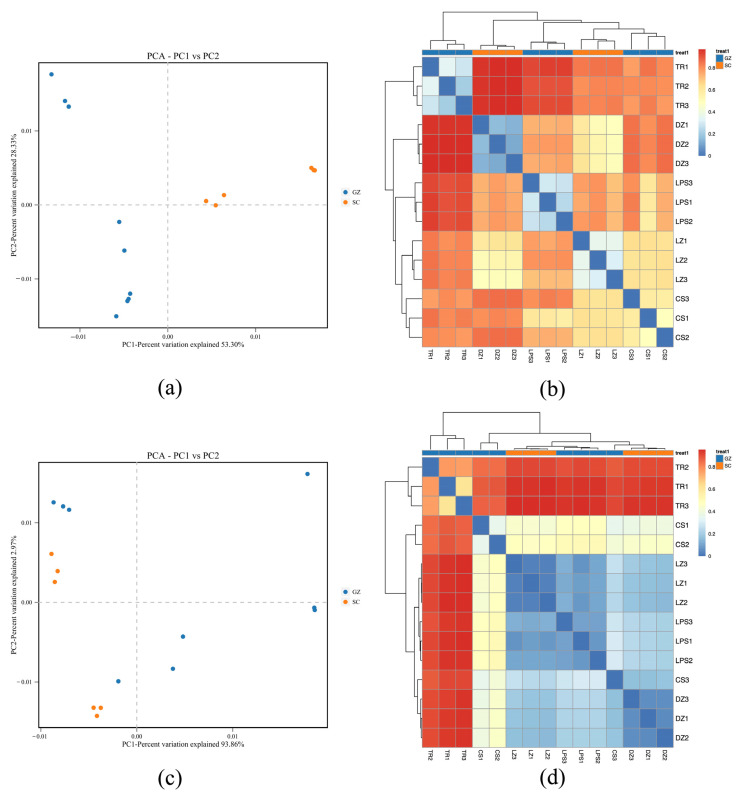
Principal component analysis (PCA) (**a**,**c**) and heatmap plot (**b**,**d**) display Bray–Curtis dissimilarity. Red and blue colors in PCA plot denote SC (Sichuan) and GZ (Guizhou) sausages, respectively. Blue indicates high similarity (low distance), and red indicates low similarity (high distance) between microbial communities of sausage samples.

PCA was conducted to assess microbial community divergence between sausage samples (Figure 3). Regarding bacterial communities, PC1 and PC2 explained 53.30% and 28.33% of the total variance, respectively. The Sichuan samples exhibited dispersion along PC1 but clustered centrally along PC2, with distinct separation from the Guizhou samples (Figure 3a). Fungal PCA revealed a pronounced variance explained by PC1 (93.86%) and PC2 (2.97%). Similarly, the Sichuan fungal communities clustered tightly along PC1, whereas the Guizhou samples showed broader dispersion (Figure 3c).

Differentiation and similarities among the five sausage samples were further analyzed based on heatmap clustering. Bacterial heatmap analysis suggested close similarity between samples LZ and CS, which clustered distinctly from DZ. Sample TR exhibited marked divergence from the other four samples (Figure 3b). Similarly, the fungal heatmap results highlighted that sample TR was obviously distinct from the remaining four sausages, whereas DZ, LZ, and LPS formed a cohesive cluster, which was reflected by the blue color in Figure 3d, suggesting shared fungal community features among these three samples.

### 3.5. LEfSe Analysis

**Figure 4:**
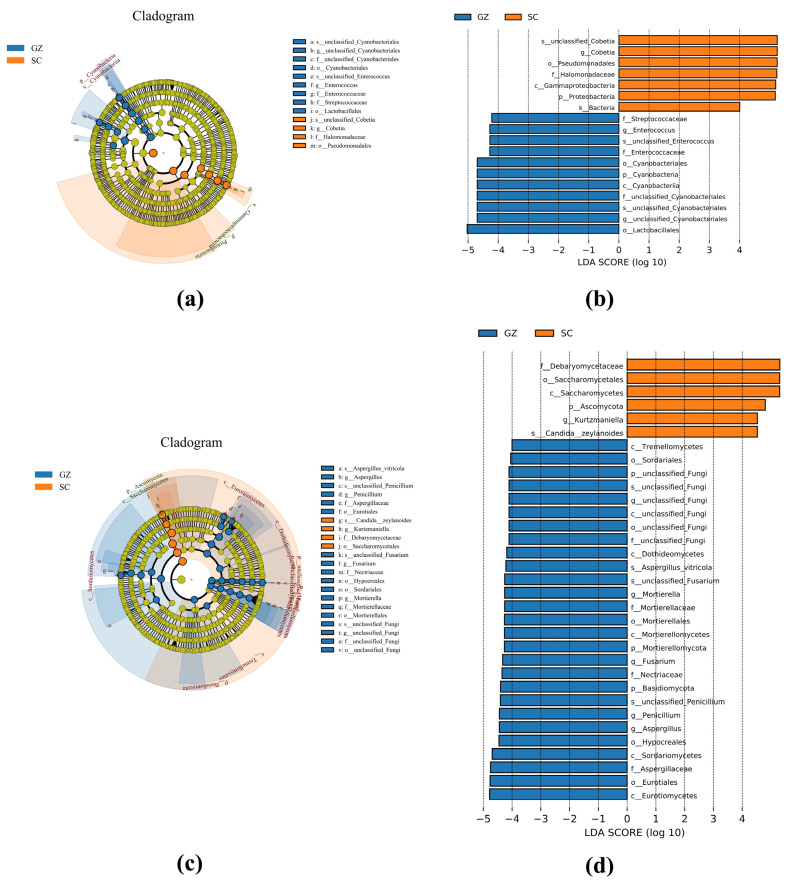
LEfSe analysis of bacteria (**a**,**b**) and fungi (**c**,**d**) of two sausage groups. Groups SC and GZ with red and blue colors represent Sichuan and Guizhou sausage samples, respectively.

Linear discriminant analysis Effect Size (LEfSe), combined with cladogram analysis, was employed to identify microbial biomarkers distinguishing Sichuan and Guizhou sausages (Figure 4). The bacterial biomarkers of the Sichuan sausages were mainly composed of *Cobetia* and *Halomonadaceae*. In contrast, *Enterococcus*, *Streptococcaceae*, Cyanobacteria, and *Lactobacillales* were considered biomarkers of the Guizhou sausages (Figure 4a,b). The fungal biomarkers further highlighted that yeasts *Debaryomycetaceae*, Saccharomycetales, *Kurtzmaniella*, and *Candida zeylanoides* were the main biomarkers of the Sichuan sausages. However, the biomarkers of the Guizhou sausages were mainly composed of filamentous molds, such as *Aspergillus*, *Mortierella*, *Fusarium*, and *Penicillium* (Figure 4c,d).

### 3.6. Correlation Analysis

**Figure 5:**
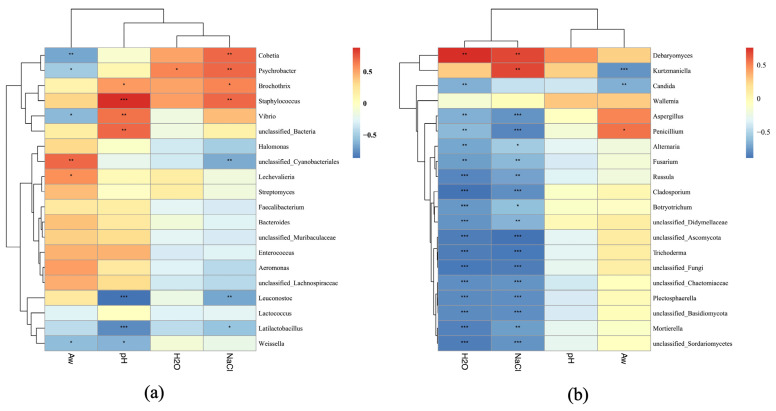
Correlation analysis between physiochemical parameters and dominant bacteria (**a**) and fungi (**b**) from five sausage samples based on Spearman’s algorithm. “*”, “**”, “***” denote significances at the *p* = 0.05, *p* = 0.01, *p* = 0.001 levels, respectively.

To further reveal the effects of physiochemical parameters on microbial composition, Spearman’s correlation analysis was conducted between four physicochemical parameters (NaCl, pH, Aw, and moisture) and the top 20 dominating bacterial and fungal taxa (Figure 5). Interestingly, the NaCl content showed positive correlations with the genera *Staphylococcus* and *Cobetia*, indicating their adaptation to high-salinity conditions. However, NaCl exhibited negative correlations with unclassified Cyanobacteriales, *Leuconostoc*, and *Latilactobacillus*, suggesting inhibitory effects on Cyanobacteria and LAB. Likewise, pH was positively correlated with *Vibrio* and *Brochothrix* as well as *Staphylococcus*, while it was negatively correlated with LAB (*Latilactobacillus*, *Leuconostoc*, and *Weissella*). Aw was positively associated with unclassified Cyanobacteriales, *Enterobacter*, and *Aeromonas* (Figure 5a). Furthermore, both NaCl and moisture exhibited positive correlations with *Debaryomyces* and *Kurtzmaniella* but negative correlations with filamentous molds including *Aspergillus*, *Penicillium*, *Mortierella*, and *Fusarium*. The positive correlation between moisture and *Debaryomyces* implied that the growth of *Debaryomyces* in sausages is favored by high humidity. Factor Aw showed positive correlations with *Penicillium*, *Aspergillus*, and *Fusarium*, etc., indicating that high water activity facilitates the proliferation of these filamentous molds (Figure 5b).

## 4. Discussion

Although the number of sausages was relatively lower and the flavor profile of the sausages was not tested, the present study performed a comparative analysis of the microbial communities of Sichuan and Guizhou sausages at the inter-provincial level. Beneficial and harmful microorganisms were preliminarily revealed. Meanwhile, the combination of the correlation results and geographical information unveiled the factors modifying the microbial composition of sausages.

Beneficial microorganisms, including halotolerant taxa (*Staphylococcus*, *Cobetia*, *Debaryomyces*) and acid-producing bacteria (LAB), exhibited distinctive distribution in Sichuan and Guizhou sausages. These functional microbiota played critical roles in enzyme excretion and flavor development. Consistent with prior studies [18,19], *Firmicutes* and *Proteobacteria* were the predominant phyla in both Sichuan and Guizhou sausages. A recent study showed that *Proteobacteria* the most predominant in Sichuan sausages, followed by *Firmicutes.* For Cantonese sausages, the dominating phyla included *Firmicutes* (68.4–80.8%) and *Proteobacteria* (13.2–18.0%) [20]. In terms of abundance, our findings showed higher values in Cantonese sausages than in Sichuan sausages. At the genus level, *Staphylococcus* and LAB (*Latilactobacillus*, *Leuconostoc*, *Weissella*, and *Lactococcus*) were ubiquitously detected in all sausage samples. It is well known that *Staphylococcus* and LAB are considered functional microorganisms in meat products during the fermentation and ripening periods [21]. *Staphylococcus sp*., such as *S. xylosus* and *S. carnosus*, can excrete protease and lipase to produce polypeptides, free amino acids, and free fatty acids, leading to the synthesis of flavor substances [22,23]. Nitrate-reductase in *S. simulans* and *S. carnosus* contributed to redness [24]. *S. xylosus* exhibited amine oxidase activity, degrading histamine and other biogenic amines [16,25]. This kind of functional genus was not only found in sausages but also other fermented meat products such as dry-curing ham [26], Sichuan smoked bacon [27], and Chinese sour meat [28]. With regard to LAB, *Weissella* is regarded as a functional LAB because of its useful metabolites such as acids and bacteriocins, which not only contribute to the formation of flavor compounds but also inhibit the growth of pathogens and spoilage microbes. *Lactobacillus* and *Lactococcus* were responsible for flavors production in sausages due to protease activity [29]. Hu et al. (2020) observed *Leuconostoc* dominance in Northeastern Chinese sausages, reinforcing its prevalence across regional styles and supporting our current findings in Guizhou [30]. In terms of application aspect, starters, consisting of *Staphylococcus* and LAB, reshaped the microbial composition and were facilitated by the production of the desired on-odors, accordingly influencing the quality of sausages [16,31]. For example, inoculation of the starter including *Lactobacillus sakei M2* and *Staphylococcus xylosus Y4* significantly increased the contents of volatile compounds such as heptanal, octanal, 2-pentanone, and 1-octen-3-ol [32].

Traditionally, the halophilic genus *Cobetia* inhabits marine environments with high-salinity. Interestingly, *Cobetia* was obtained from Sichuan sausages characterized by an elevated NaCl content (>5%), mirroring its reported presence in other high-salt fermented meats such as Chinese traditional bacon and dry-cured ham [33,34]. *Cobetia* contributes to flavor development through enzymatic activity and metabolite synthesis. In Mianning ham, this genus promoted the formation of flavors such as benzaldehyde, 3-methylthio-propanal, trans-2-nonenal, and (E,E)-2,4-decadienal [35,36]. Meanwhile, it also secreted a variety of extracellular hydrolases, such as amylase, lipase, protease, and nuclease, and performed higher hydrolysis activity under high-salinity conditions [37]. *Cobetia* was positively correlated with five dipeptides and four glycerophospholipids in Chinese bacon [34], which not only contributed to umami flavor but also facilitated biofilm formation via the production of extracellular proteins and polysaccharides. Biofilm further helps *Cobetia* adapt to high-salt environments like Sichuan sausages [38]. However, the metabolic pathways of *Cobetia* in meat matrices remain poorly characterized.

Concerning the fungal community in fermented meats, yeasts produce a wide range of esters, higher alcohols, carbonyl compounds, and fatty acid derivatives [39,40]. Molds synthesize volatile compound precursors and flavors as a result of lipolytic and proteolytic activities [41]. Consistent with prior studies on dry sausages [42,43], dominant fungal genera in our study included *Debaryomyces*, *Kurtzmaniella*, *Aspergillus*, *Fusarium*, *Penicillium*, and *Mortierella*. *Debaryomyces* sp. (e.g., *D. hansenii*) is indispensable for the fermentation and ripening of dry sausages. This halotolerant yeast stabilizes the redness of fermented sausages due to its ability to degrade peroxides [44]. Regardless of simple in vitro models or complex sausage models, *D. hansenii* could synthesize volatile compounds in fermented sausages, including esters, acids, branched alcohols, and aldehydes, thus shaping the final volatile profile due to its proteolytic and lipolytic activity [40,45]. As presented in Figure 5, a positive correlation between moisture content and *Debaryomyces* suggested that the growth of *D. hansenii* in sausage is favored by high humidity, which was not in agreement with a previous finding reported by Bonaïti [46].

*Kurtzmaniella zeylanoides* (formerly *Candida zeylanoides)*, a psychrotrophic yeast previously identified in Chinese traditional fermented fish [47], Italian fermented fish sausage [48], and Portuguese cacholeira blood sausage [49], exhibited lipolytic activity [50] and produced flavor compounds such as benzene ethanol and 3-methyl-1-butanol [51]. Furthermore, in the dry fermented sausage models, strains including *D. hansenii* SH4 and *K. C. zeylanoides* DQ7 showed significantly positive correlations with volatiles (acetic acid, hexanoic acid, ethanol, phenethyl alcohol, ethyl acetate, and ethyl hexanoate) [52], underscoring their metabolic versatility.

Regarding potential pathogens and spoiling bacteria, since Chinese smoke-cured sausages are produced in an open environment under spontaneous fermentation, Cyanobacteria, *Enterococcus*, *Psychrobacter*, *Brochothrix*, *Faecalibacterium*, *Aeromonas*, and *Vibrio* are generally introduced into sausages during the fermentation and ripening stages. Typically, Cyanobacteria originate from water, soil, or environment. The occurrence of Cyanobacteria was caused by hand-making procedures or unsanitary conditions [15]. *Enterococcus faecium* has previously been found and isolated from dry fermented sausages [14,19,53]. This species possibly transferred antibiotic resistance genes to *Listeria monocytogenes* [54]. Meanwhile, *E. faecium* and *E. faecalis* produced biogenic amines in dry fermented sausages [55]. *Brochothrix*, *Psychrobacter*, *Aeromonas*, *Serratia*, *Pseudomonas*, and *Streptococcus* were detected from fermented sausages in different regions of China and recognized as spoilage bacteria due to the production of off-odors and off-flavors [19,53]. Overall, Chinese dry fermented sausages are highly susceptible to undesirable pathogenic and spoilage bacteria. In contrast, opportunistic pathogenic and spoilage bacteria merely exist in western sausages due to the use of starters, which was supported by a comparative analysis result [18]. In addition, the inoculation of starters strongly inhibited undesired microorganisms (e.g., *Yersinia*, *Enterobacter*, *Acinetobacter*, *Psychrobacter)* and off-flavor substances [16,31].

With respect to adverse effects, many filamentous fungi showed an atoxigenic character, while some potential mycotoxin-producing fungi belonged to genera *Penicillium*, *Aspergillus* and *Fusarium*, such as *Penicillium nordicum*, *P. olsonii*, *P. expansum*, *P. viridicatum*, *P. granulatum*, *P. oxalicum*, *P. commune*, *Aspergillus versicolor*, *A. fischeri*, *A. ochraceus*, and *Aspergillus carbonarius* [56]. For instance, mycotoxin compound ochratoxin A poses a great risk to human’s health and was primarily produced by *P. nordicum* in the high Aw condition [57]. *Cladosporium oxysporum* and *Penicillium* spp. caused another undesired effect—food spoilage—resulting in the production of off-odors and unpleasant taste [58]. Recently, it was shown that autochthonous microorganism *Debaryomyces hansenii* and *Staphylococcus xylosus* in fermented meat products can significantly inhibit the growth of *P. nordicum* and accordingly reduce the production of ochratoxin A [59,60]. Genera *Staphylococcus* and *Debaryomyces* were predominant in the Sichuan and Guizhou sausages, except sample TR, where the abundances of *Penicillium* and *Aspergillus* were very low, suggesting a clear inhibitory effect (Figure 2b,d). Meanwhile, *Staphylococcus* and *Debaryomyces* are possibly developed as potential sausage starters due to the capacity of flavor production and their antagonistic effect of harmful fungi. With regard to *Fusarium*, previous studies demonstrated that mycotoxins zearalenone and fumonisins were mainly produced by *Fusarium culmorum* and *Fusarium graminearum*. These mycotoxins were distributed in food and feed, posing a risk to animal and human health due to their global propagation and serious economic loss [61,62].

As suggested previously, differences in the microbial communities of other fermented foods, such as traditional Sichuan bacon [63], Tibetan yak jerky [15], and Xinjiang Kazak cheese [64], were attributed to the raw materials, climate conditions, and processing methods. In this case study, from a geographic perspective, although both cities belong to Sichuan Province, Luzhou city is far from Dazhou city, and the climate conditions in winter are different between these two cities. Conversely, Luzhou city and Chishui county are very close, and their climate conditions are also similar (Figure 1). However, the microbial communities, especially the fungal communities, of samples LZ and DZ were highly similar, while the distinction between LZ and CS was significant (Figure 2 and Figure 3). The four physiochemical parameters of samples DZ and LZ were approximate. However, Aw, the water content, and NaCl content of sample CS were much lower than those of two Sichuan sausages (Table 1). Accordingly, the argument that differences in microbial community of sausages between Sichuan sausages and Guizhou sausages are possibly caused by processing conditions such as the addition of salt rather than climate factors is made.

## 5. Conclusions

In conclusion, a comparative analysis of the bacterial and fungal communities in Sichuan and Guizhou sausages was conducted in the present study despite limitations in sample size and flavor profile characterization. The dominating bacteria *Staphylococcus* and *Cobetia*, and yeasts *Debaryomyces*, *Kurtzmaniella* mainly inhabited Sichuan sausages. However, acid-tolerant lactic acid bacteria (*Lactococcus*, *Leuconostoc*, *Weissella*, and *Lactobacillus*) and molds (*Aspergillus*, *Mortierella*, *Fusarium*, and *Penicillium*) were largely distributed in Guizhou sausages, especially TR. Integrative analysis of geographic, climatic, and physiochemical data suggested that the discrepancy in the microbial composition of sausages from Sichuan and Guizhou provinces was possibly attributed to the processing conditions (salt addition) rather than climate. Key beneficial microbiota (e.g., *Staphylococcus*, *Debaryomyces*, *Cobetia*, LAB) and potential harmful microbes (Cyanobacteria, *Enterococcus*, *Penicillium*, *Aspergillus* and *Fusarium*) were identified, which are facilitated by the development of novel starters composed of defined functional microorganisms. Subsequently, isolating functional strains and characterizing flavor profiles are required in further studies.

## Figures and Tables

**Table 1:** Physicochemical parameters of Sichuan sausages and Guizhou sausages.

Sample ID ^1^	pH	H_2_O (%)	Aw	NaCl (%)
DZ	5.97 ± 0.062 ^b^	23.95 ± 0.071 ^b^	0.805 ± 0.004 ^c^	5.60 ± 0.067 ^a^
LZ	5.68 ± 0.174 ^c^	27.96 ± 0.067 ^a^	0.818 ± 0.004 ^bc^	5.13 ± 0.089 ^b^
CS	5.94 ± 0.086 ^b^	14.15 ± 0.173 ^e^	0.761 ± 0.016 ^d^	2.87 ± 0.156 ^d^
LPS	6.22 ± 0.012 ^a^	23.13 ± 0.324 ^c^	0.865 ± 0.002 ^a^	3.60 ± 0.133 ^c^
TR	5.51 ± 0.073 ^c^	19.83 ± 0.324 ^d^	0.830 ± 0.007 ^b^	2.27 ± 0.111 ^e^

^1^: DZ, Dazhou; LZ, Luzhou; CS, Chishui; LPS, Liupanshui; TR, Tongren. Letters following values show significant differences. These five sausage samples were measured in triplicate.

**Table 2:** α-diversity indices of bacterial and fungal community of Sichuan and Guizhou fermented sausages.

Sample ID ^1^	ACE		Chao1		Simpson		Shannon		PD Whole Tree	
	B	F	B	F	B	F	B	F	B	F
DZ	480.2 ± 55.9 ^c^	350.5 ± 21.1 ^c^	475.1 ± 54.5 ^c^	338.6 ± 21.6 ^c^	0.75 ± 0.01 ^c^	0.4 ± 0.02 ^c^	3.38 ± 0.16 ^c^	1.54 ± 0.14 ^c^	68.9 ± 15.4 ^c^	81.2 ± 1.3 ^b^
LZ	1627.4 ± 332.5 ^ab^	257.3 ± 8.1 ^c^	1622.5 ± 334.1 ^ab^	246.1 ± 17.1 ^c^	0.92 ± 0.02 ^a^	0.17 ± 0.02 ^d^	6 ± 0.64 ^a^	0.7 ± 0.1 ^c^	185 ± 26.8 ^b^	62.7 ± 3.7 ^b^
CS	1617.5 ± 113.2 ^ab^	768.9 ± 261.1 ^a^	1603.8 ± 114.3 ^ab^	767.4 ± 259.4 ^a^	0.91 ± 0.04 ^a^	0.67 ± 0.14 ^b^	5.81 ± 0.45 ^a^	4.15 ± 1.33 ^b^	203.8 ± 19.5 ^b^	168.3 ± 45.8 ^a^
LPS	1793.7 ± 83.8 ^a^	465.1 ± 47 ^bc^	1785.4 ± 84.4 ^a^	457.5 ± 47.4 ^bc^	0.93 ± 0.01 ^a^	0.21 ± 0.05 ^d^	6.12 ± 0.15 ^a^	1.21 ± 0.3 ^c^	313.8 ± 20.1 ^a^	114.2 ± 23.4 ^ab^
TR	1337.3 ± 93.2 ^b^	704.1 ± 201.5 ^ab^	1327.6 ± 94.1 ^b^	703.6 ± 201.9 ^ab^	0.85 ± 0.04 ^b^	0.96 ± 0.03 ^a^	4.75 ± 0.41 ^b^	7.04 ± 0.79 ^a^	200.5 ± 5.7 ^b^	162.7 ± 42.6 ^a^

^1^: DZ, Dazhou; LZ, Luzhou; CS, Chishui; LPS, Liupanshui; TR, Tongren. B, bacteria; F, fungi. Letters following values show significant differences. These five sausage samples were measured in triplicate.

## Data Availability

The original contributions presented in this study are included in the article, the raw sequences data can be acquired in SRA database with accession No. PRJNA870249. Further inquiries can be directed to the corresponding authors.

## Abbreviations

The following abbreviations are used in this manuscript

LAB	Lactic acid bacteria
HTS	High-throughput sequencing
ITS	Internal transcribed spacer
OTU	Operational taxonomic unit
PCA	Principal component analysis

## References

[1] Oliveira M., Ferreira V., Magalhães R., Teixeira P. (2018). Biocontrol strategies for Mediterranean-style fermented sausages. Food Res. Int..

[2] Flores M., Piornos J.A. (2021). Fermented meat sausages and the challenge of their plant-based alternatives: A comparative review on aroma-related aspects. Meat Sci..

[3] Wang Z., Wang Z., Ji L., Zhang J., Zhao Z., Zhang R., Bai T., Hou B., Zhang Y., Liu D. (2021). A review: Microbial diversity and function of fermented meat products in China. Front. Microbiol..

[4] Cocolin L., Dolci P., Rantsiou K., Urso R., Cantoni C., Comi G. (2009). Lactic acid bacteria ecology of three traditional fermented sausages produced in the North of Italy as determined by molecular methods. Meat Sci..

[5] Lewis W.H., Tahon G., Geesink P., Sousa D.Z., Ettema T.J.G. (2021). Innovations to culturing the uncultured microbial majority. Nat. Rev. Microbiol..

[6] Pham V.H.T., Kim J. (2012). Cultivation of unculturable soil bacteria. Trends Biotechnol..

[7] Blaiotta G., Pennacchia C., Ercolini D., Moschetti G., Villani F. (2003). Combining denaturing gradient gel electrophoresis of 16S rDNA V3 region and 16S-23S rDNA spacer region polymorphism analyses for the identification of staphylococci from Italian fermented sausages. Syst. Appl. Microbiol..

[8] Cocolin L., Manzano M., Aggio D., Cantoni C., Comi G. (2001). A novel polymerase chain reaction (PCR)—Denaturing gradient gel electrophoresis (DGGE) for the identification of *Micrococcaceae* strains involved in meat fermentations. Its application to naturally fermented Italian sausages. Meat Sci..

[9] Albano H.C.P., Henriques I., Correia A.C.M., Hogg T.A., Teixeira P. (2008). Characterization of microbial population of ‘Alheira’ (a traditional Portuguese fermented sausage) by PCR-DGGE and traditional cultural microbiological methods. J. Appl. Microbiol..

[10] Fontana C., Vignolo G., Cocconcelli P.S. (2005). PCR-DGGE analysis for the identification of microbial populations from Argentinean dry fermented sausages. J. Microbiol. Methods.

[11] Lu S., Ji H., Wang Q., Li B., Li K., Xu C., Jiang C. (2015). The effects of starter cultures and plant extracts on the biogenic amine accumulation in traditional Chinese smoked horsemeat sausages. Food Control.

[12] Wang Y., Li B., Liu Y., Huang X., Zhang N., Yang Y., Xiao Z., Yu Q., Chen S., He L. (2021). Investigation of diverse bacteria encoding histidine decarboxylase gene in Sichuan-style sausages by culture-dependent techniques, polymerase chain reaction–denaturing gradient gel electrophoresis, and high-throughput sequencing. Lebensm.-Wiss. Technol..

[13] Wang C., Liu H., He L., Li C. (2022). Determination of bacterial community and its correlation to volatile compounds in Guizhou Niuganba, a traditional Chinese fermented dry-cured beef. Lebensm.-Wiss. Technol..

[14] Wang X., Wang S., Zhao H. (2019). Unraveling microbial community diversity and succession of Chinese Sichuan sausages during spontaneous fermentation by high-throughput sequencing. J. Food Sci. Technol..

[15] Wen R., Lv Y., Li X., Chen Q., Kong B. (2021). High-throughput sequencing approach to reveal the bacterial diversity of traditional yak jerky from the Tibetan regions. Meat Sci..

[16] Ren H., Deng Y., Wang X. (2022). Effect of a compound starter cultures inoculation on bacterial profile and biogenic amine accumulation in Chinese Sichuan sausages. Food Sci. Hum. Wellness.

[17] Chen X., Li J., Zhou T., Li J., Yang J., Chen W., Xiong Y. (2016). Two efficient nitrite-reducing *Lactobacillus* strains isolated from traditional fermented pork (Nanx Wudl) as competitive starter cultures for Chinese fermented dry sausage. Meat Sci..

[18] Wang X., Zhang Y., Ren H., Zhan Y. (2018). Comparison of bacterial diversity profiles and microbial safety assessment of salami, Chinese dry-cured sausage and Chinese smoked-cured sausage by high-throughput sequencing. Lebensm.-Wiss. Technol..

[19] Zhang Q.Q., Li D., Zhang W., Jiang M., Chen X.H., Dong M.S. (2021). Comparative analysis of the bacterial diversity of Chinese fermented sausages using high-throughput sequencing. Lebensm.-Wiss. Technol..

[20] Chen X., Yan F., Qu D., Wan T., Xi L., Hu C.Y. (2024). Aroma characterization of Sichuan and Cantonese sausages using electronic nose, gas chromatography–mass spectrometry, gas chromatography-olfactometry, odor activity values and metagenomic. Food Chem. X.

[21] Laranjo M., Potes M.E., Elias M. (2019). Role of starter cultures on the safety of fermented meat products. Front. Microbiol..

[22] Wang H., Li Y., Xia X., Liu Q., Sun F., Kong B. (2022). Flavour formation from hydrolysis of pork meat protein extract by the protease from *Staphylococcus carnosus* isolated from Harbin dry sausage. Lebensm.-Wiss. Technol..

[23] Wang Y., Li F., Chen J., Sun Z., Wang F., Wang C., Fu L. (2021). High-throughput sequencing-based characterization of the predominant microbial community associated with characteristic flavor formation in Jinhua Ham. Food Microbiol..

[24] Gøtterup J., Olsen K., Knøchel S., Tjener K., Stahnke L.H., Møller J.K.S. (2008). Colour formation in fermented sausages by meat-associated staphylococci with different nitrite- and nitrate-reductase activities. Meat Sci..

[25] Wang X., Ren H., Wang W., Zhang Y., Bai T., Li J., Zhu W. (2015). Effects of inoculation of commercial starter cultures on the quality and histamine accumulation in fermented sausages. J. Food Sci..

[26] Yang Z., Wu Y., Wang G., Zhou N., Zhao S., Chen G., Zheng Z., Ren R., Liao G. (2025). Comparative analysis of physicochemical properties and microbial community structure in five types of Yunnan dry-cured hams. Lebensm.-Wiss. Technol..

[27] Song Z., Cao Y., Zhang Y., Zhang Z., Shi X., Zhang W., Wen P. (2022). Effects of storage methods on the microbial community and quality of Sichuan smoked bacon. Lebensm.-Wiss. Technol..

[28] Zang J., Li T., Liu K., Wu J., Zhang Z., Liu X., Zhao J., Peng C., Li Z. (2025). Correlations between microbiota succession and volatile profiles development and biogenic amine formation involved in the ripening of Chinese sour meat. Lebensm.-Wiss. Technol..

[29] Hu Y., Tian Y., Zhu J., Wen R., Chen Q., Kong B. (2022). Technological characterization and flavor-producing potential of lactic acid bacteria isolated from traditional dry fermented sausages in northeast China. Food Microbiol..

[30] Hu Y., Zhang L., Liu Q., Wang Y., Chen Q., Kong B. (2020). The potential correlation between bacterial diversity and the characteristic volatile flavour of traditional dry sausages from Northeast China. Food Microbiol..

[31] Xiao Y., Liu Y., Chen C., Xie T., Li P. (2020). Effect of *Lactobacillus plantarum* and *Staphylococcus xylosus* on flavour development and bacterial communities in Chinese dry fermented sausages. Food Res. Int..

[32] Ma Y., Gao Y., Xu Y., Zhou H., Zhou K., Li C., Xu B. (2023). Microbiota dynamics and volatile metabolite generation during sausage fermentation. Food Chem..

[33] Mu Y., Su W., Mu Y., Jiang L. (2019). Combined application of high-throughput sequencing and metabolomics reveals metabolically active microorganisms during Panxian ham processing. Front. Microbiol..

[34] Wang Y., Wang Z., Han Q., Xie Y., Zhou H., Zhou K., Li X., Xu B. (2022). Comprehensive insights into the evolution of microbiological and metabolic characteristics of the fat portion during the processing of traditional Chinese bacon. Food Res. Int..

[35] Zeng Q., Ji L., Wang W., Zhang J., Bai T., Gan L., Chen L. (2025). Comparative studies on physicochemical properties, volatile flavor substances and microbial community of Mianning ham at different altitudes. Front. Microbiol..

[36] Huang Y., Wang Z., Gan L., Zhang J., Wang W., Ji L., Chen L. (2024). Study on the changes and correlation of microorganisms and flavor in different processing stages of Mianning ham. Foods.

[37] Dang H., Zhu H., Wang J., Li T. (2009). Extracellular hydrolytic enzyme screening of culturable heterotrophic bacteria from deep-sea sediments of the Southern Okinawa Trough. World J. Microbiol. Biotechnol..

[38] Flemming H.C., Wuertz S. (2019). Bacteria and archaea on Earth and their abundance in biofilms. Nat. Rev. Microbiol..

[39] Dzialo M.C., Park R., Steensels J., Lievens B., Verstrepen K.J. (2017). Physiology, ecology and industrial applications of aroma formation in yeast. FEMS Microbiol. Rev..

[40] Flores M., Corral S., Cano-García L., Salvador A., Belloch C. (2015). Yeast strains as potential aroma enhancers in dry fermented sausages. Int. J. Food Microbiol..

[41] Sunesen L.O., Stahnke L.H. (2003). Mould starter cultures for dry sausages-selection, application and effects. Meat Sci..

[42] Belleggia L., Ferrocino I., Reale A., Haouet M.N., Corvaglia M.R., Milanović V., Boscaino F., Di Renzo T., Di Bella S., Borghi M. (2022). Unravelling microbial populations and volatile organic compounds of artisan fermented liver sausages manufactured in Central Italy. Food Res. Int..

[43] Wen R., Sun F., Li X.-A., Chen Q., Kong B. (2021). The potential correlations between the fungal communities and volatile compounds of traditional dry sausages from Northeast China. Food Microbiol..

[44] Chao H., Yen Y., Ku M.S.B. (2009). Characterization of a salt-induced DhAHP, a gene coding for alkyl hydroperoxide reductase, from the extremely halophilic yeast *Debaryomyces hansenii*. BMC Microbiol..

[45] Murgia M.A., Marongiu A., Aponte M., Blaiotta G., Deiana P., Mangia N.P. (2019). Impact of a selected *Debaryomyces hansenii* strain’s inoculation on the quality of Sardinian fermented sausages. Food Res. Int..

[46] Bonaïti C., Leclercq-Perlat M.N., Latrille E., Corrieu G. (2004). Deacidification by *Debaryomyces hansenii* of smear soft cheeses ripened under controlled conditions: Relative humidity and temperature influences. J. Dairy Sci..

[47] Zang J., Xu Y., Xia W., Yu D., Gao P., Jiang Q., Yang F. (2018). Dynamics and diversity of microbial community succession during fermentation of Suan yu, a Chinese traditional fermented fish, determined by high throughput sequencing. Food Res. Int..

[48] Belleggia L., Ferrocino I., Rita Corvaglia M., Cesaro C., Milanović V., Cardinali F., Garofalo C., Cocolin L., Aquilanti L., Osimani A. (2022). Profiling of autochthonous microbiota and characterization of the dominant lactic acid bacteria occurring in fermented fish sausages. Food Res. Int..

[49] Belleggia L., Ferrocino I., Reale A., Boscaino F., Di Renzo T., Corvaglia M.R., Cocolin L., Milanović V., Cardinali F., Garofalo C. (2020). Portuguese cacholeira blood sausage: A first taste of its microbiota and volatile organic compounds. Food Res. Int..

[50] Mendoza L.M., Padilla B., Belloch C., Vignolo G. (2014). Diversity and enzymatic profile of yeasts isolated from traditional llama meat sausages from north-western Andean region of Argentina. Food Res. Int..

[51] Mi R., Chen X., Xiong S., Qi B., Li J., Qiao X., Chen W., Qu C., Wang S. (2021). Predominant yeasts in Chinese Dong fermented pork (Nanx Wudl) and their aroma-producing properties in fermented sausage condition. Food Sci. Hum. Wellness.

[52] Wen R., Yin X., Hu Y., Chen Q., Kong B. (2022). Technological properties and flavour formation potential of yeast strains isolated from traditional dry fermented sausages in Northeast China. Lebensm.-Wiss. Technol..

[53] Huang Z., Shen Y., Huang X., Qiao M., He R.K., Song L. (2021). Microbial diversity of representative traditional fermented sausages in different regions of China. J. Appl. Microbiol..

[54] Jahan M., Holley R.A. (2016). Transfer of antibiotic resistance from *Enterococcus faecium* of fermented meat origin to *Listeria monocytogenes* and *Listeria innocua*. Lett. Appl. Microbiol..

[55] Komprda T., Sládková P., Petirová E., Dohnal V., Burdychová R. (2010). Tyrosine- and histidine-decarboxylase positive lactic acid bacteria and enterococci in dry fermented sausages. Meat Sci..

[56] Sidari R., Tofalo R. (2024). Dual role of yeasts and filamentous fungi in fermented sausages. Foods.

[57] Delgado J., Rondan J.J., Nunez F., Rodriguez A. (2021). Influence of an industrial dry-fermented sausage processing on ochratoxin A production by *Penicillium nordicum*. Int. J. Food Microbiol..

[58] Lozano-Ojalvo D., Rodriguez A., Cordero M., Bernaldez V., Reyes-Prieto M., Cordoba J.J. (2015). Characterisation and detection of spoilage mould responsible for black spot in dry-cured fermented sausages. Meat Sci..

[59] Roncero E., Andrade M.J., Álvarez M., Cebrián E., Rodríguez M. (2024). *Debaryomyces hansenii* reduces ochratoxin A production by *Penicillium nordicum* on dry-cured ham agar through volatile compounds. Lebensm.-Wiss. Technol..

[60] Cebrián E., Núñez F., Álvarez M., Roncero E., Rodríguez M. (2022). Biocontrol of ochratoxigenic *Penicillium nordicum* in dry-cured fermented sausages by *Debaryomyces hansenii* and *Staphylococcus xylosus*. Int. J. Food Microbiol..

[61] Damiano S., Longobardi C., Ferrara G., Piscopo N., Riccio L., Russo V., Meucci V., De Marchi L., Esposito L., Florio S. (2023). Oxidative status and histological evaluation of wild boars’ tissues positive for zearalenone contamination in the campania region, southern Italy. Antioxidants.

[62] Hartinger T., Grabher L., Pacífico C., Angelmayr B., Faas J., Zebeli Q. (2022). Short-term exposure to the mycotoxins zearalenone or fumonisins affects rumen fermentation and microbiota, and health variables in cattle. Food Chem. Toxicol..

[63] Wang S., Wang X., Pan W., Liu A., Liu S., Yang Y., Zou L. (2021). Evaluation of Bacterial Diversity and Quality Features of Traditional Sichuan Bacon from Different Geographical Region. Appl. Sci..

[64] Zheng X., Liu F., Li K., Shi X., Ni Y., Li B., Zhuge B. (2018). Evaluating the microbial ecology and metabolite profile in Kazak artisanal cheeses from Xinjiang, China. Food Res. Int..

